# Functional differences in hepatitis C virus nonstructural (NS) 3/4A- and 5A-specific T cell responses

**DOI:** 10.1038/srep24991

**Published:** 2016-05-04

**Authors:** Fredrik Holmström, Margaret Chen, Anangi Balasiddaiah, Matti Sällberg, Gustaf Ahlén, Lars Frelin

**Affiliations:** 1 Department of Laboratory Medicine, Division of Clinical Microbiology, F68, Karolinska Institutet, Karolinska University Hospital Huddinge, S-141 86 Stockholm, Sweden; 2 Department of Dental Medicine, Karolinska Institutet, Huddinge, S-141 04 Stockholm, Sweden

## Abstract

The hepatitis C virus nonstructural (NS) 3/4A and NS5A proteins are major targets for the new direct-acting antiviral compounds. Both viral proteins have been suggested as modulators of the response to the host cell. We have shown that NS3/4A- and NS5A-specific T cell receptors confer different effector functions, and that killing of NS3/4A-expressing hepatocytes is highly dependent on IFN-γ. We here characterize the functional differences in the T cell responses to NS3/4A and NS5A. NS3/4A- and NS5A-specific T cells could be induced at various frequencies in wild-type-, NS3/4A-, and NS5A-transgenic mice. Priming of NS5A-specific T cells required a high DNA dose, and was unlike NS3/4A dependent on both CD4^+^ and CD8^+^ T cells, but less influenced by CD25^+^/GITR^+^ regulatory T cells. The presence of IL-12 greatly improved specific CD8^+^ T cell priming by NS3/4A but not by NS5A, suggesting a less dependence of IFN-γ for NS5A. This notion was supported by the observation that NS5A-specific T cells could eliminate NS5A-expressing hepatocytes also in the absence of IFN-γ-receptor-2. This supports that NS3/4A- and NS5A-specific T cells become activated and eliminate antigen expressing, or infected hepatocytes, by distinct mechanisms, and that NS5A-specific T cells show an overall less dependence of IFN-γ.

The hepatitis C virus (HCV) is a global health problem with 130–170 million individuals chronically infected worldwide and it is estimated that 2 million people are newly infected each year^1^. The disease progresses silently from a clinical perspective and with time the infection may cause fibrosis, cirrhosis and an increased risk for hepatocellular carcinoma^2^,^3^.

The introduction of direct-acting antivirals (DAA) has revolutionized the treatment of chronic HCV infection with sustained virological responses (SVR) above 90 percent^4^,^5^,^6^. However, despite the high cure rate in patients there is still some obstacle to be solved. Firstly, the DAAs are associated with high costs and is a difficult issue not only for resource-poor countries where a majority of all chronic HCV carriers already lives but also for many high-income countries that only can prioritize certain patients groups. Secondly, although based on existing knowledge, no absolute contra-indications to the DAAs approved in the EU region exist today^7^, caution is required for several patient groups (e.g. DAA experienced patients who failed earlier treatment, patients with renal impairment, liver transplanted patients, patient with hepatic decompensation, children and pregnant women)^8^. Lastly, DAA treatment does not protect against re-infection^9^. Activation of post-cure HCV-specific immune responses are hence of importance to reduce the risk of re-infection. An effective immunity against HCV should also benefit non-responder patients, patients that developed DAA resistance and patients who discontinued treatment due to side effects.

The HCV NS3/4A and NS5A proteins are major targets for the new DAAs^4^,^10^,^11^. The NS3/4A protein complex is well characterized with helicase and protease activities^12^. In addition, the NS3/4A complex has also been shown to interfere with innate and adaptive immune responses in order to maintain chronicity^13^. The NS5A protein is an important component of the HCV replication machinery and for virion assembly^14^,^15^. However, we still lack a complete understanding about NS5A and its functions. Previous data have shown that NS5A modulates the host immune response by protecting hepatocytes from cytolytic killing^16^. Moreover, we have previously shown that a codon-optimized NS5A-DNA vaccine efficiently primed polyfunctional NS5A-specific CD8^+^ T cells in both wild-type- and immune- tolerant NS5A-transgenic (Tg) mice^17^.

In this study we compared the T cell responses to HCV NS3/4A and NS5A with the aim of better understanding the immune modulatory role of NS5A during immune priming and effector functions.

## Results

### Priming of NS5A-specific T cells

**Figure 1:**
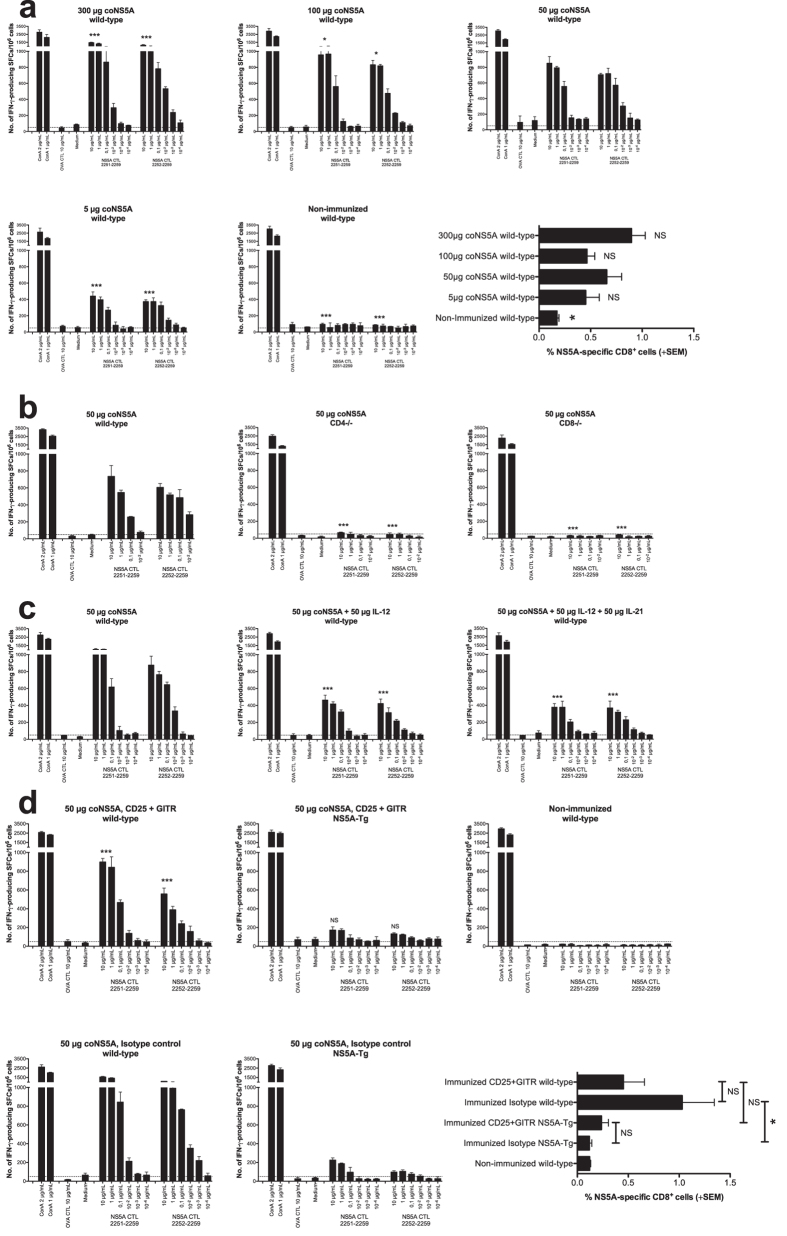
Immunological components affecting priming of NS5A-specific T cell responses. Groups of five wild-type (**a**–**d**), CD4^−/−^ (**b**), CD8^−/−^ (**b**), and NS5A-Tg (**d**) mice were immunized once with indicated doses intramuscularly (i.m.) with coNS5A-pVAX1 followed by *in vivo* EP. One group of mice was left untreated. Two weeks after immunization the mice were sacrificed and splenocytes harvested for determination of T cell responses. A comparison of the number of IFN-γ spot forming cells (SFCs) by ELISpot assay after stimulation with indicated antigens was done in immunized and non-immunized groups of mice. Results are given as the mean SFCs/10^6^ (+SD) splenocytes with a cutoff set at 50 SFCs/10^6^ splenocytes. In (**c**) the coNS5A-pVAX1 plasmid was delivered in combination with 50 μg mIL-12-pORF1 or in combination of 50 μg mIL-12-pORF1 and 50 μg mIL-21-pORF1. In (**d**) coNS5A-pVAX1 immunization was carried out in CD25^+^/GITR^+^ depleted wild-type- and NS5A-Tg mice or the same groups given isotype controls. In (a and d) the expansion of NS5A-specific CD8^+^ T cells in wild-type (a and d) and NS5A-Tg (**d**) mice was determined using direct *ex vivo* pentamer staining. VILDSFDPL epitope-specific CD8^+^ T cells are shown as the percentage of NS5A-pentamer positive CD8^+^ T cells (+SEM). The statistical difference shown (ELISpot), indicate a statistical difference from the group of wild-type (**a**–**d**) or NS5A-Tg (**d**) mice immunized with 50 μg coNS5A-pVAX1 (*p < 0.05, and ***p < 0.001, by comparing area under the curve (AUC) and analysis of variance (ANOVA)). The statistical difference (frequencies of NS5A-specific CD8^+^ T cells) between the groups is indicated as *p < 0.05 determined by the Mann-Whitney U test. NS = not significant.

We have previously shown that NS5A-specific CD8^+^ T cells producing IFN-γ and IL-2 can be primed in both wild-type and NS5A-transgenic (Tg) mice^17^. To compare the T cell priming of NS5A with NS3/4A we immunized mice with NS5A-DNA doses ranging from 300 μg to 5 μg. This revealed that, unlike NS3/4A, the priming of NS5A-specific T cell responses required much higher doses as compared to NS3/4A (Fig. 1a, and data not shown^18^, and Levander *et al*., submitted for publication). At least 50 μg NS5A-DNA was needed to prime a potent NS5A-specific T cell response, and higher DNA doses induced stronger T cell responses (Fig. 1a, p < 0.001 and p < 0.05, AUC and ANOVA). In all further experiments the 50 μg vaccine dose was used if not stated otherwise.

### Cell types affecting priming of NS5A-specific T cells

We have previously shown that the priming of NS3/4A-specific T cell responses is independent of CD4^+^ T cells but dependent on CD8^+^ T cells, and that the T cell priming in NS3/4A-Tg mice can be greatly improved by depletion of Tregs^19^. We wanted to test whether this was true also in the priming of NS5A-specific T cells. Unlike HCV NS3/4A, we found that both CD4^+^ and CD8^+^ T cells were important for the priming NS5A-specific T cells determined by IFN-γ production (Fig. 1b) and quantification of CD8^+^ T cells (data not shown). We next investigated if co-expression of one or more cytokine genes could improve priming of NS5A-specific T cells. Murine (m) interleukin (IL)-12 expressing plasmids have demonstrated to improve the priming of IFN-γ producing T cells^20^,^21^, and IL-21 has been suggested to promote proliferation and effector functions of virus-specific T cells^22^,^23^,^24^. Unexpectedly, neither of the cytokines improved IFN-γ or IL-2 production nor increased frequencies of NS5A-specific CD8^+^ T cells. In fact, the presence of these cytokines seemed to even lower immune responses (Fig. 1c, and data not shown). This is quite distinct from the priming of NS3/4A-specific T cells.

It is well known that HCV-specific T cell responses are dysfunctional during chronic HCV infection^25^, and this dysfunction is actively maintained until HCV replication is blocked^26^. Both Tregs and PD-1 expression has been proposed as participants in this dysfunction. We therefore asked whether depletion of CD25^+^ and GITR^+^ Tregs could improve NS5A-specific T cell priming in wild-type and NS5A-Tg mice. Depletion of Tregs did not improve IFN-γ and IL-2 production or the frequency of primed NS5A-specific CD8^+^ T cells (Fig. 1d). In contrast, the depletion of Tregs even decreased IFN-γ and IL-2 production and frequencies of NS5A-specific CD8^+^ T cells in wild-type mice (Fig. 1d). This is in pronounced contrast to the priming of NS3/4A-specific T cells, which was greatly improved by depletion of Tregs^27^.

### NS3/4A- and NS5A-specific T cell activation in the presence of a dysfunctional T cell response to HCV

**Figure 2:**
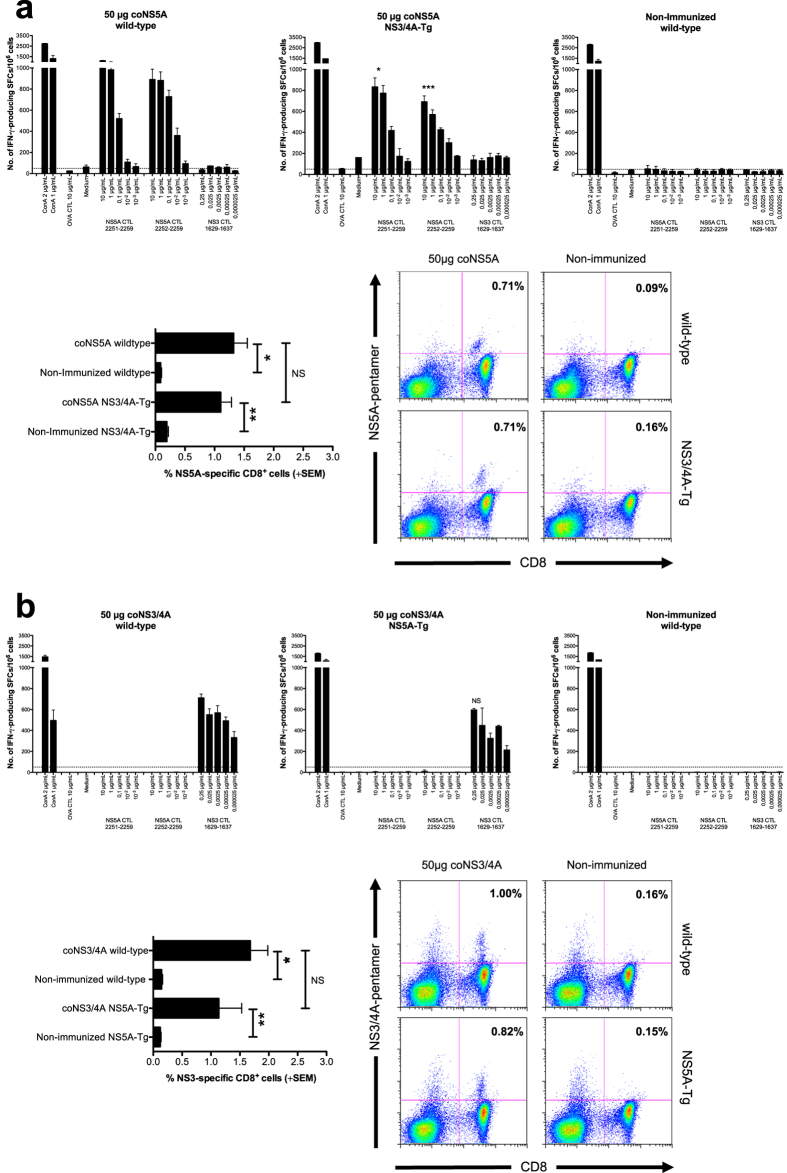
NS3/4A- and NS5A-specific T cell activation in the presence of a dysfunctional T cell response to HCV. Groups of five wild-type (**a**,**b**), NS3/4A-Tg (**a**), and NS5A-Tg (**b**) mice were immunized once with 50 μg coNS5A-pVAX1 or coNS3/4A-pVAX1 intramuscularly (i.m.) followed by *in vivo* EP. One group of mice was left untreated. Two weeks after immunization the mice were sacrificed and splenocytes harvested for determination of T cell responses. A comparison of the number of IFN-γ spot forming cells (SFCs) by ELISpot assay after stimulation with indicated antigens was done in immunized and non-immunized groups of mice. Results are given as the mean SFCs/10^6^ (+SD) splenocytes with a cutoff set at 50 SFCs/10^6^ splenocytes. The statistical difference shown, indicate a statistical difference from the group of wild-type mice immunized with 50 μg coNS5A-pVAX1 or coNS3/4A-pVAX1 (*p < 0.05, and ***p < 0.001, by AUC and ANOVA). NS = not significant. In (a and b) the expansion of NS3- or NS5A-specific CD8^+^ T cells in wild-type (**a**,**b**), NS3/4A-Tg (**a**), and NS5A-Tg (**b**) mice was determined using direct *ex vivo* pentamer staining. GAVQNEVTL (NS3) or VILDSFDPL (NS5A) epitope-specific CD8^+^ T cells are shown as the percentage of NS3- or NS5A-pentamer positive CD8^+^ T cells (+SEM). Also, representative dot plots from each group are shown. The statistical difference between the groups is indicated as *p < 0.05, and **p < 0.01 determined by the Mann-Whitney U test. NS = not significant.

We have used NS3/4A-Tg mice as a model for human HCV infection, as the dysfunctional HCV-specific T cells in the Tg mice share some features of the T cells seen in chronically infected humans^17^,^27^. To better understand the T cell function in the NS3/4A-Tg and NS5A-Tg mice, we analyzed the priming of NS5A- or NS3/4A-specific T cell responses. We first found a slightly lower, albeit significant, levels of NS5A-specific IFN-γ production in NS3/4A-Tg mice compared to immunized wild-type mice (Fig. 2a, p < 0.001, and p < 0.05, AUC and ANOVA), whereas no differences were seen when comparing NS3/4A-specific immune responses primed in wild-type and NS5A-Tg mice (Fig. 2b, p = NS). Similar frequencies of NS3- and NS5A-specific CD8^+^ T cells were obtained when immunizing wild-type and Tg mice with NS3/4A- or NS5A-DNA (Fig. 2a,b). Also, representative dot plots of flow cytometry analysis are shown (Fig. 2a,b). In addition, NS3/4A-specific T cell priming was similar in NS3/4A/5A-Tg mice as previously shown in NS3/4A-Tg^27^ (data not shown).

### Presence of heterologous T cells at the site of priming improves NS5A-specific T cell activation

**Figure 3:**
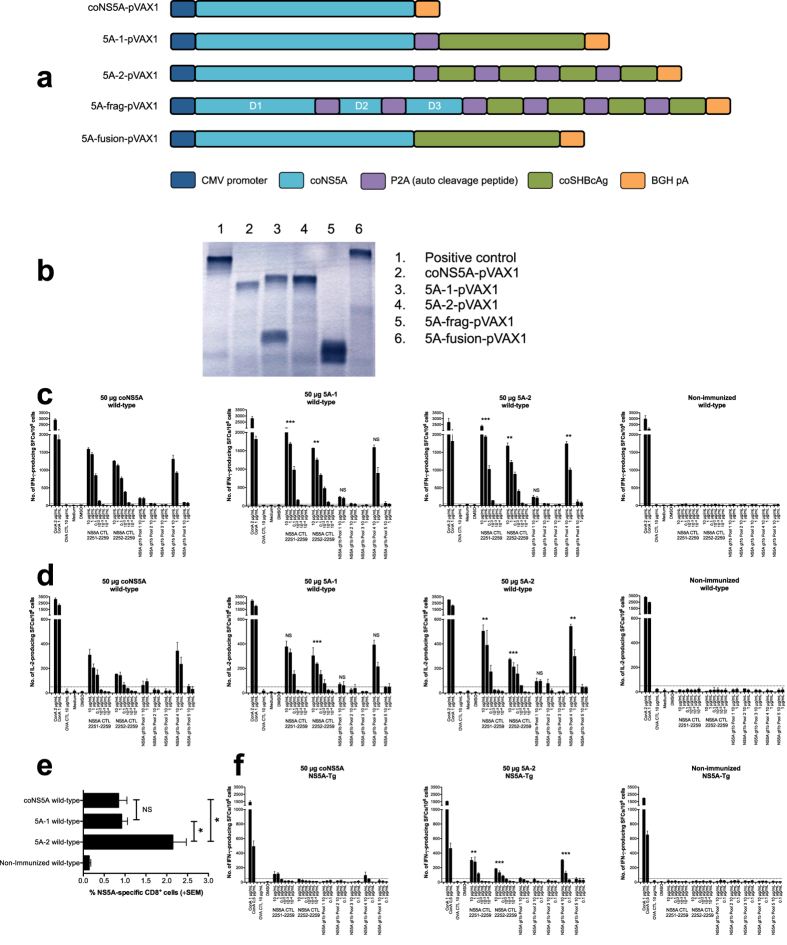
Presence of heterologous T cells at the site of priming improves NS5A-specific T cell activation. Design of the coNS5A-coStork-HBcAg containing DNA constructs evaluated in the present study (**a**). The plasmids encode a codon-optimized HCV NS5A protein followed by different variants of HBcAg derived from stork, either as full-length, fragmented or as fusion constructs. All constructs (e.g. coNS5A, 5A-1, 5A-2, 5A-frag, and 5A-fusion) were tested for their expression by an *in vitro* transcription and translation assay (**b**). The positive control consists of a Luciferase-expression plasmid producing a protein of 61 kDa. Groups of five wild-type- (**c**–**e**) and NS5A-Tg (**f**) mice were immunized once with 50 μg of indicated plasmids in a volume of 30 μL by intramuscular immunization using the IVIN technology followed by *in vivo* EP. One group of mice was left untreated. Two weeks after immunization the mice were sacrificed and splenocytes harvested for determination of T cell responses. A comparison of the number of IFN-γ (**c**,**f**) and IL-2 (**d**) spot forming cells (SFCs) by ELISpot assay after stimulation with indicated antigens was done in immunized and non-immunized groups of mice. Results are given as the mean SFCs/10^6^ (+SD) splenocytes with a cutoff set at 50 SFCs/10^6^ splenocytes. The statistical difference shown, indicate a statistical difference from the group of wild-type or NS5A-Tg mice immunized with 50 μg coNS5A-pVAX1 (**p < 0.01, and ***p < 0.001, by AUC and ANOVA). NS = not significant. In (**e**) the expansion of NS5A-specific CD8^+^ T cells in wild-type mice was determined using direct *ex vivo* pentamer staining. VILDSFDPL epitope-specific CD8^+^ T cells are shown as the percentage of NS5A-pentamer positive CD8^+^ T cells (+SEM). The statistical difference between the groups is indicated as *p < 0.05 determined by the Mann-Whitney U test. NS = not significant.

To further define the differences in the priming of NS3/4A- and NS5A-specific T cells we evaluated the ability of heterologous T cells to support and improve priming of NS5A-specific T cells. Hence, we designed four fusion constructs of NS5A and stork-HBcAg (Fig. 3a). Based on previous experience we know that the use of stork or human HBcAg as a heterologous antigen effectively adjuvant priming of NS3/4A-specific T cells^27^ (Levander *et al*., submitted for publication). Importantly, it should be highlighted that stork and human HBcAg share less than 40% amino acid homology^28^. When comparing the amino acid sequence of four previously identified HLA-A2.1 and H-2^b^ epitopes we found no evident sequence homology (Levander *et al*., submitted for publication). Thus, the epitopes are most likely not cross-reactive on a T cell level. All fusion constructs were confirmed to have the expected size and cleavage pattern (Fig. 3b). We now found that the presence of heterologous T cells improved IFN-γ and IL-2 production from NS5A-specific T cells (Fig. 3c,d, and data not shown, p < 0.001, and p < 0.01, AUC and ANOVA), and expansion of NS5A-specific CD8^+^ T cells (Fig. 3e, and data not shown, p < 0.05, Mann-Whitney U test). This was true also in the immune-tolerant NS5A-Tg mice with a dysfunctional T cell response to NS5A (Fig. 3f, p < 0.001, and p < 0.01, AUC and ANOVA). Thus, the presence of heterologous T cells effectively adjuvant the priming of both NS3/4A- and NS5A-specific T cells in the presence of a naive or a dysfunctional HCV-specific immune response.

**Figure 4:**
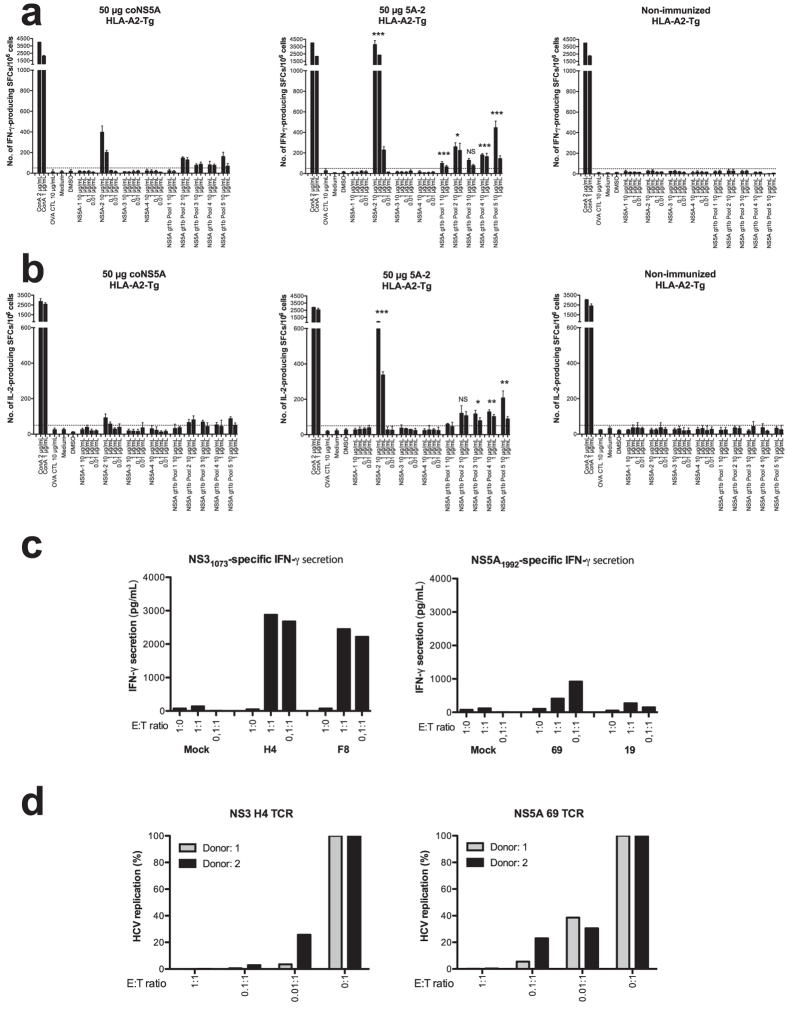
Presence of heterologous T cells improves priming of HLA-A2-restricted NS5A-specific T cells and anti-HCV activity by HCV TCR-redirected human T cells. Groups of five HHD mice were immunized once with 50 μg of indicated plasmids in a volume of 30 μL by intramuscular immunization using the IVIN technology followed by *in vivo* EP. One group of mice was left untreated. Two weeks after immunization the mice were sacrificed and splenocytes harvested for determination of T cell responses. A comparison of the number of IFN-γ (**a**) and IL-2 (**b**) spot forming cells (SFCs) by ELISpot assay after stimulation with indicated antigens was done in immunized and non-immunized groups of mice. Results are given as the mean SFCs/10^6^ (+SD) splenocytes with a cutoff set at 50 SFCs/10^6^ splenocytes. The statistical difference shown, indicate a statistical difference from the group of HHD mice immunized with 50 μg coNS5A-pVAX1 (*p < 0.05, **p < 0.01, and ***p < 0.001, by AUC and ANOVA). NS = not significant. (**c**) IFN-γ secretion from H4 and F8 (NS3 TCR) and 69 and 19 (NS5A TCR) redirected human T cells co-cultured overnight with peptide (NS3_1073_ gt1a or NS5A_1992_ gt1b)-loaded T2 cells or controls at effector:target (E:T) ratio 1:0, 1:1, and 0.1:1. (**d**) Anti-HCV effect on Huh7^A2^HCV^Rep^ replicon cells conferred by indicated TCR-redirected human T cells (donor 1 and 2) at indicated E:T ratios 1:1, 0.1:1, 0.01:1, and 0:1. Values are expressed as percent HCV replication. Huh7^A2^HCV^Rep^ replicon cells alone were set to 100% HCV replication).

We also evaluated the role of heterologous adjuvant sequences in a mouse strain that express the human HLA-A2.1 molecule (e.g. HHD-C57BL/6J^29^. We found with respect to a human HLA-A2-restricted NS5A-specific response, that the presence of heterologous T cells at the priming site improved the NS5A-specific T cell response (Fig. 4a,b, p < 0.001, p < 0.01 and p < 0.05, AUC and ANOVA). The CD8^+^ T cell response primarily focused to the VLTDFKTWL_1992–2000_ CTL epitope (Fig. 4 and Table 1). Polymorphism within this CTL epitope was rare when comparing published GenBank isolate sequences (Table 2). Thus, heterologous sequences effectively promote the priming of HLA-A2-restricted NS5A-specific response.

We then further compared the functional properties of HLA-A2-restricted NS3- and NS5A-specific T cells using human T cells transfected by T cell receptor (TCR) mRNA to provide equal expression level and frequency (>90% transfection efficiency) of NS3- respectively NS5A-specific TCRs on human T cells from HCV RNA negative human donors. We have previously shown that NS3- and NS5A-specific TCRs differ in their functional properties^30^. We here compared their ability to produce IFN-γ and to inhibit HCV RNA replication. The NS3-specific T cells produced high levels of IFN-γ and the TCR-transfected T cells from two different donors effectively inhibited HCV RNA replication (Fig. 4c,d). In contrast, the NS5A-specific TCRs produced low levels of IFN-γ, and inhibited HCV RNA replication less efficiently (Fig. 4c,d). Thus, NS3-specific T cells are highly antiviral whereas NS5A-specific T cells are less so both *in vivo* and *in vitro*.

### The immune molecules required for the clearance of NS5A-expressing hepatocytes are distinct from those required to clear NS3/4A-expressing cells

**Figure 5:**
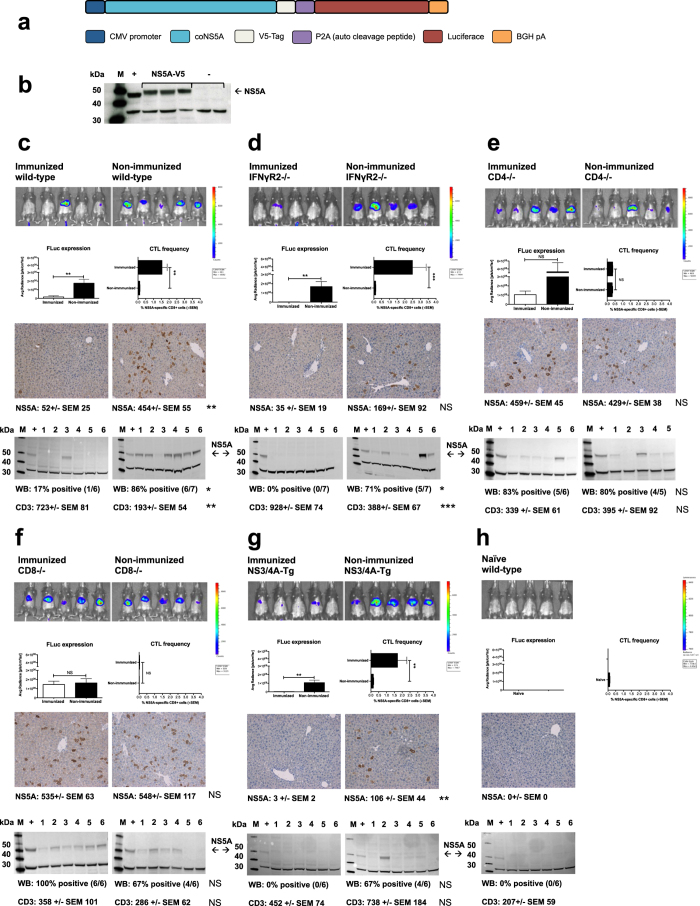
The immune molecules required for the clearance of NS5A-expressing hepatocytes are distinct from those required to clear NS3/4A-expressing cells. Design of the coNS5A-V5-Luc-pcDNA3.1(−) plasmid used for hydrodynamic injection (**a**). In (**b**) wild-type mice received a hydrodynamic injection of the coNS5A-V5-Luc-pcDNA3.1(−) plasmid (lane 3–5, indicated with “NS5A-V5”) or left-untreated (lane 6–7, indicated with “−”). “M” indicates the molecular weight marker (e.g. MagicMark, Life Technologies, Carlsbad, CA). “+” indicates positive control, a NS5A-Tg mouse liver homogenate. In (**c**–**g**) the biodistribution of NS5A-luciferase determined by using *in vivo* imaging (Caliper Life Sciences, Hopkinton, MA) is shown in immunized and non-immunized wild-type, IFNγR2^−/−^, CD4^−/−^, CD8^−/−^, and NS3/4A-Tg mice (5–7 mice/group) 72 hours after NS5A-luciferase transfection of hepatocytes. Immunizations were performed as described in Fig. 3. One naïve group was included to determine the background in the *in vivo* imaging and immunological assays. Statistical difference has been indicated (**p < 0.01) determined by the Mann-Whitney U test. NS = not significant. VILDSFDPL epitope-specific CD8^+^ T cells are shown (**c**–**h**) as the percentage of NS5A-pentamer positive CD8^+^ T cells (+SEM). The statistical difference between the groups is indicated as **p < 0.01, and ***p < 0.001 determined by the Mann-Whitney U test. NS = not significant. Detection of NS5A-protein in livers of mice 72 hours after hydrodynamic injection was determined by immunohistochemistry and Western blot analysis. “M” indicates the molecular weight marker (e.g. MagicMark, Life Technologies, Carlsbad, CA). “+” indicates positive control, a liver homogenate from a wild-type mouse that was hydrodynamically injected with the coNS5A-V5-Luc-pcDNA3.1(−) plasmid. CD3^+^ T cell infiltration of livers was determined by immunohistochemistry. Statistical differences in NS5A-expression and CD3^+^ T cell infiltration by immunohistochemistry were determined by Mann-Whitney U test (**p < 0.01, and ***p < 0.001, NS = not significant), and NS5A by western blot analysis determined by Fischer’s exact test (*p < 0.05, NS = not significant). Representative *in vivo* imaging, histological and western blot pictures are shown.

Clearance of HCV NS3/4A-expressing hepatocytes *in vivo* has been shown to require CD8^+^ T cells and IFNγ but not CD4^+^ T cells^31^. As the priming of NS5A-specific T cells had different requirements as compared to NS3-specific T cells, we analyzed if this was also reflected on effector functions *in vivo*. To investigate these basic questions we applied the hydrodynamic challenge model where mice with specific genetic alteration were made transiently transgenic for NS5A. Mice received a hydrodynamic tail vein injection of an NS5A-luciferase-expression construct (Fig. 5a). Groups of mice was either immunized or left non-immunized prior to the hydrodynamic injection. At 72 hours post the hydrodynamic injection mice were monitored for luciferase-expression, thereafter sacrificed and livers collected for western blot analysis and immunohistochemistry. Immune responses were determined by IFN-γ and IL-2-production, and quantification of NS5A-specific CD8^+^ T cells (Fig. 5, and data not shown). The NS5A-luciferase-expression construct was shown to be functional evidenced by the presence of an NS5A-protein band in mice receiving a hydrodynamic injection (Fig. 5b). NS5A-luciferase activity and NS5A-expressing cells disappeared quicker in immunized compared to non-immunized wild-type, IFNγR2^−/−^, and NS3/4A-Tg mice, supporting a hepatic trafficking and *in vivo* functionality of the primed T cells, and that these factors had no or little impact on T cell effector function (Fig. 5c,d,g, p < 0.01, Mann-Whitney U test). Consistent with this had immunized mice higher frequencies of NS5A-specific CD8^+^ T cells as compared to non-immunized mice (Fig. 5c,d,g, p < 0.001, and p < 0.01, Mann-Whitney U test). Immunized mice had also higher numbers of intrahepatic CD3-positive cells as compared to the non-immunized groups (Fig. 5c,d,g, p < 0.01 (wild-type mice), p < 0.001 (IFNγR2^−/−^ mice), and p = NS (NS3/4A-Tg mice)). Interestingly, there was no difference between NS5A-luciferase expression in immunized and non-immunized CD4^−/−^ and CD8^−/−^ mice, suggesting that both these cell types were essential for elimination of NS5A-expressing cells (Fig. 5e,f). Thus, much unlike NS3-specific T cells, intrahepatic NS5A-specific T cell effector function relies on both CD4^+^ and CD8^+^ T cells, but not on IFNγR2.

## Discussion

T cells have a central role in the control and elimination of the HCV infection. A recent study showed that when the viral replication has been suppressed by DAAs the HCV-specific T cell response is at least partly restored^26^. Thus, there is an unexpectedly profound suppression of the HCV replication on the host immune system in the ongoing infection. Thus, even in the era of DAAs it is inevitable that we will need to understand the role of T cells during HCV infection. The aim of this study was to better understand how NS3/4A and NS5A interacts with host T cells with respect to the priming and effector functions to provide a better understanding of any immune modulatory role exerted by these proteins. The so far least well understood protein is NS5A. The NS5A protein has been shown to be important for both RNA replication and virus assembly^14^,^15^. The NS5A protein is known to interact with multiple components of the host cell^32^,^33^, and to modulate the host cell immunity^16^,^34^,^35^. In this study we focused on immunological characteristics of HCV NS5A and NS3/4A.

To understand the basic characteristics of NS5A T cell priming we investigated the importance of immunogen dose, effects of cytokines and heterologous sequences, and CD4^+^ and CD8^+^ T cells during immunization. NS3/4A is well known to modulate the host response in both humans and mice, but despite that NS3/4A does prime T cells at low DNA doses, is easily adjuvanted by IL-12 or heterologous sequences. Furthermore, priming of CD8^+^ T cells as well as effector functions are independent of CD4^+^ T cells^19^. In contrast to NS3/4A, the priming of NS5A-specific T cells was highly dependent on high immunogen dose, was not adjuvanted by IL-12 or IL-21, but by the presence of heterologous T cells. Also, both CD4^+^ and CD8^+^ T cells were required for NS5A-specific CD8^+^ T cell priming and effector function. This is distinct from NS3/4A. However, we do not know if NS5A could be adjuvanted by these cytokines when combined with heterologous sequences (e.g. stork-HBcAg).

Patients with chronic HCV infection are known to have exhausted and dysfunctional T cells, and this dysfunction can be restored by effective inhibition of the HCV replication^26^. The T cell dysfunction has been proposed to be actively maintained by Tregs or expression of various inhibitory molecules^36^,^37^. We have shown that Tregs profoundly affect the priming of NS3/4A-specific CD8^+^ T cells^27^. We therefore depleted CD25^+^/GITR^+^ Tregs prior to priming NS5A-specific T cells in wild-type and NS5A-Tg mice. In contrast to NS3/4A, the NS5A-specific T cell priming was not improved by depletion of Tregs. Thus, our results indicate no major role of CD25^+^/GITR^+^ Tregs during NS5A T cell priming, at least not in this model. This merits further investigation, especially the effects of blocking/depleting other inhibitory molecules or cell components.

We finally addressed the functionality of intrahepatic T cell responses. Both NS5A- and NS3/4A-specific T cell responses could be raised in the respective transgenic mouse strain. However, lower NS5A-specific T cell responses were recorded in the NS3/4A-Tg mice, suggesting that hepatic NS3/4A-expression may influence priming of NS5A-specific T cells. The mechanism behind this is not clear and merits further investigation. DNA primed *in vivo* functional NS5A-specific CD8^+^ T cell responses were shown in wild-type-, IFNγR2^−/−^, and NS3/4A-Tg mice, evidenced by clearance of hepatic NS5A by *in vivo* imaging and immunochemistry. Both CD4^+^ and CD8^+^ T cell responses were required for the clearance of hepatic NS5A-expression. Thus, the activation and function of NS3/4A- and NS5A-specific T cells differ^31^,^38^.

In conclusion, the functional aspects of NS3/4A- and NS5A-specific T cells differ with respect to both activation and effector functions. NS3/4A-specific T cells are easily activated independent of CD4^+^ help and by low vaccine doses, are effectively improved by cytokines inducing IFN-γ, and are inhibited by Tregs. Elimination of NS3/4A-expressing hepatocytes is dependent on CD8^+^ and IFNγR2, but not CD4^+^ T cells. Priming of NS5A-specific T cells is dependent on both CD4^+^ and CD8^+^ T cells, unaffected by IL-12 or Tregs, but improved by heterologous sequences/T cells. Clearance of NS5A-expressing hepatocytes requires both CD4^+^ and CD8^+^ T cells but not IFNγR2. Many of these differences are consistent with NS3/4A-specific T cells having higher avidities and producing higher levels of IFN-γ than NS5A-specific T cells, as shown previously and herein, respectively. Both these types of cells may be required in an effective polyclonal T cell response to HCV that eliminate HCV infected cells during DAA therapy.

## Methods

### Animals

Inbred C57BL/6J (H-2^b^), NS5A-transgenic (Tg), NS3/4A-Tg, NS3/4A/5A-Tg, HHD-transgenic for HLA-A2.1, IFNγR2^−/−^, CD4^−/−^ and CD8^−/−^ on C57BL/6J (H-2^b^) background have been used within this study. The animals were housed at Karolinska Institutet, Division of Comparative Medicine, Clinical Research Center, Karolinska University Hospital, Huddinge, Sweden. Female mice at the age of 6–10 weeks (at the start of the experiment) were caged (5–10 mice per cage) and fed with a commercial diet (RM3 (p) IRR diet; Special Diet Service). All handling and experimental protocol involving animals were approved by the Ethical Committee for Animal Research at Karolinska Institutet. The methods were carried out in accordance with the approved guidelines. The animals were either purchased (Charles River Laboratories, Sulzfeld, Germany and the Jackson Laboratory, Bar Harbor, ME) or bred in-house. C57BL/6J mice transgenic for HCV NS5A genotype 1b have liver-specific expression under control of the mouse albumin promoter and enhancer. The NS5A transgene construct also contains a rabbit β-globuline intron and V5 tag followed by a SV40 polyadenylation signal^16^,^17^. C57BL/6J mice transgenic for HCV NS3/4A genotype 1a have liver-specific expression under the control of the mouse major urinary protein (MUP) promoter^39^. The NS3/4A/5A-Tg mice were obtained by crossing the NS3/4A-Tg and NS5A-Tg mice. Inbred HHD (HHD^+^ H-2D^b−/−^ β2m^−/−^) mice transgenic for HLA-A2.1 monochain histocompatibility class I molecule^29^ were used to study HLA-A2-restricted CD8^+^ T cell responses. All NS5A-Tg and NS3/4A-Tg mice were genotyped by PCR to verify the transgene. Extraction of DNA from the tail was performed using the DNeasy Blood and Tissue Kit (Qiagen, Hilden, Germany), according to the manufacturer’s instructions, and the transgenes (e.g. NS5A and NS3/4A) and the reference gene tubulin were amplified by PCR using the REDExtract-N-Amp^™^ Tissue PCR Kit (Sigma-Aldrich, St. Louis, MO).

### PBMC from healthy blood donors

Blood samples were collected from HCV RNA negative blood donors at Karolinska University Hospital. Peripheral blood mononuclear cells (PBMCs) were isolated using Ficoll-Hypaque density gradient centrifugation. Ethical permission was obtained from the Regional Ethical Review Board (EPN) in Stockholm. Informed consent was obtained from all subjects. All experimental protocols were approved by the Regional Ethical Review Board (EPN) in Stockholm and the methods were carried out in accordance with the approved guidelines. HLA-A2 typing was performed by flow cytometry.

### Cell lines

The T2 cell line (HLA A2.1^+^) was maintained in RPMI 1640 medium supplemented with 10% fetal bovine serum (FBS) (Sigma Aldrich, St Louis, MO), 2 mM L-glutamine, 10 mM HEPES, 100 U ml^−1^ penicillin, 100 μg ml^−1^ streptomycin.

The Huh-7-Lunet cell line designated Lunet-HLA-A2-luc/neoET (Huh7^A2^HCV^Rep^) has been described previously^30^. The Lunet-HLA-A2-luc/neoET has ectopic HLA-A2 expression and contains a selectable HCV sub-genomic RNA replicon of genotype 1b (Con1-ET) that also expresses the firefly luciferase gene fused to the selectable marker by ubiquitin. The Lunet-HLA-A2-luc/neoET cell line was maintained in Dulbecco’s modified Eagle’s medium supplemented with 10% fetal bovine serum (FBS) (Sigma Aldrich, St Louis, MO), 2 mM L-glutamine, 100 U ml^−1^ penicillin, 100 μg ml^−1^ streptomycin, 1 mM nonessential amino acids, and puromycin (1 μg/ml) and G418 (0.5 mg/ml) (Sigma Aldrich). All cells were grown in a humidified 37 °C and 5% CO_2_ incubator.

### Peptides and Proteins

In order to study HCV-specific CD8^+^ T cell responses, previously identified MHC class I/HLA-A2 epitopes were used in the immunological assays. The following MHC class I peptides were used; HCV NS5A-CTL (VILDSFDPL, aa 2251–2259, H-2D^b^ and ILDSFDPL, aa 2252-2259, H-2K^b^)^17^, HCV NS3-CTL (GAVQNEVTL, aa 1629–1637 H-2K^b^)^40^, and HLA-A2 restricted CTL epitopes; ILDSFDPLR (aa 2252–2260)^41^, VLTDFKTWL (aa 1992–2000)^42^, LLREDVTFQV (aa 2145–2154)^42^, and SPDADLIEANL (aa 2221–2231)^43^. As negative control, the Ovalbumin CTL epitope (OVA-CTL) was used; SIINFEKL (OVA 257–264). All MHC class I/HLA-A2 peptides were synthesized by automated peptide synthesis as described previously (ChronTech Pharma AB, Huddinge, Sweden)^44^.

A total of 44 20-mer peptides (each having 10 amino acid overlap) covering the full-length HCV NS5A genotype 1b were purchased from Sigma Aldrich (St. Louis, MO). The 44 peptides were divided in five peptides pools as outlined: pool 1: peptide 1–9 (1971–2060), pool 2: 10–18 (2061–2150), pool 3: 19–27 (2151–2240), pool 4: 28–36 (2241–2330), pool 5: 37–44 (2331–2419).

Concanavalin A (ConA) were purchased from Sigma Aldrich (St. Louis, MO).

### Plasmids

The HCV NS3/4A and NS5A genes have been previously codon optimized (co) and inserted into the pVAX1 vector^17^,^40^. The coNS3/4A-pVAX1 plasmid has been described earlier^19^,^40^ (GenBank accession number: AR820945.1; http://www.ncbi.nlm.nih.gov/genbank, ChronVac-C; ChronTech Pharma AB, Stockholm, Sweden), and originates from a HCV genotype 1a virus. The coNS5A-pVAX1 plasmid has been described earlier^17^ and originates from a HCV genotype 1b virus (GenBank accession number D16435). The following plasmids 5A-1-pVAX1, 5A-2-pVAX1, 5A-frag-pVAX1 and 5A-fusion-pVAX1 are based on the coNS5A-pVAX1 plasmid, which has been modified with the addition of either full-length or fragmented gene-sequences of stork hepatitis B core antigen (stork-HBcAg) (GenBank accession number: GZ869879.1) (see Fig. 3a). The fragmented NS5A-stork-HBcAg constructs contain 2A self-cleaving peptide sequences. The 5A-1-pVAX1 construct is a coNS5A-coStork-HBcAg fusion construct containing a 2A self-cleaving peptide sequence between NS5A and stork-HBcAg. The 5A-2-pVAX1 construct is identical to 5A-1, but contain coStork-HBcAg modifications consisting of the introduction of three 2A self-cleaving peptide sites in the coStork-HBcAg gene sequence, allowing the expressed 2A peptide to cleave the modified stork-HBcAg into four parts (stork-HBcAg: aa 1–66, aa 67–132, aa 133–198, and aa 199–264). The 5A-frag-pVAX1 construct is identical to 5A-2, but contain coNS5A modifications consisting of the introduction of two 2A self-cleaving peptide sites separating the NS5A domain I and II and domain II and III. The 5A-fusion-pVAX1 construct is identical to 5A-1 except the exclusion of the 2A self-cleaving peptide sequence between NS5A and stork-HBcAg. Mouse interleukin-12 and IL-21 (pORF-mIL-12 or IL-21, mIL-12 or mIL-21) was purchased from InvivoGen (San Diego, USA).

For hydrodynamic injections, the coNS5A-V5-Luc-pcDNA3.1(−) plasmid was generated (Fig. 4a). It is based on the coNS5A gene but cloned into the pcDNA3.1(−) plasmid and with the addition of a V5-tag and the firefly luciferase (FLuc2) gene for easy detection by western blot and/or by *in vivo* imaging. The FLuc2-gene was purchased from Promega (Madison, WI). All HCV and stork-HBcAg containing plasmids were made synthetically (Retrogen, San Diego, CA and Genscript, Piscataway, NJ). Plasmids were grown in competent TOP10 *E. coli* (Life Technologies, Carlsbad, CA) and purified using Plasmid DNA Purification and QIAfilter Plasmid Mega Kit (Qiagen, Hilden, Germany), according to the manufacturer’s protocol. Purified plasmid DNA was resolved and diluted in sterile PBS and its concentration determined spectrophotometrically (Biophotometer; Eppendorf, Hamburg, Germany). Restriction enzyme digest were performed to ensure that the plasmid contains the gene of interest with the correct size. All plasmids were sequenced by Eurofins MWG Operon (Ebersberg, Germany) to verify the correct nucleotide sequence.

### *In vitro* transcription and translation assay

All expression constructs were analyzed using the *in vitro* transcription and translation assay (TNT; Promega, Madison, WI) to ensure that the inserted genes were intact and could be correctly translated. The assay was performed according to manufacture’s instructions. In brief, constructs were *in vitro* transcribed and translated for 90 minutes at 30 °C in the presence of [^35^S]-methionine (Amersham, Buckinghamshire, UK). The translated proteins were separated on a 12% SDS-PAGE gel and thereafter visualized by exposure to a Hyper film-MP (Amersham, Buckinghamshire, UK) for 48 hours and developed using a Protec Processor Optimax (Siemens Healthcare, Erlangen, Germany).

### Immunization and *in vivo* depletion protocols

Groups of mice were immunized by the intramuscular (i.m.) route using a regular needle or using the *in vivo* intracellular injection device (IVIN)^45^. All immunizations were performed in the left tibialis cranialis muscle. Immediately after the immunization the muscle were electroporated (EP) using the Cliniporator^2^ device (IGEA, Carpi, Italy) with a pulse pattern of one 1 ms 600 V/cm pulse followed by a 400 ms 60 V/cm pulse. The amount of DNA injected was between 300 μg to 5 μg diluted in volumes of 50 μL or 30 μL PBS. Mice were immunized once and sacrificed two weeks post immunization.

Groups of mice were *in vivo* depleted of cells expressing regulatory molecules by intra peritoneal (i.p.) injection of 200 μg anti-CD25 (clone PC-61.5.3; rat igG1) and 200 μg anti-GITR (clone DTA-1; rat IgG2b) every second to third day with start two weeks before immunization and continuously until mice were sacrificed. The following isotype controls were used: 200 μg anti-rat IgG1 (clone HRPN) and 200 μg anti-rat IgG2b (clone LTF-2). All antibodies were purchased from Bio X Cell (West Lebanon, NH).

Transient expression of HCV NS5A and FLuc in livers of mice was employed by a hydrodynamic injection as described previously^46^. Briefly, mice received an intravenous (i.v.) injection in the tail vein of 100 μg NS5A-V5-Luc-pcDNA3.1(−) in 1,8 mL of sterile Ringers solution. At 72 hours after the hydrodynamic injection, mice were analyzed by *in vivo* imaging for luciferase expression, thereafter sacrificed and livers collected for western blot analysis.

### Sample preparation and western blot analysis

Liver biopsies (100 mg) were collected and homogenized using sonication (Vibra Cell Homogenizator, Chemical Instruments, Lidingö, Sweden) in 1 mL radio- immunoprecipitation assay buffer (150 mM NaCl, 50 mM Tris-HCl [pH 7.4], 1 mM EDTA, 1% Triton X-100, 1% sodium deoxycholate, 1% SDS, 2 mM PMSF, 0.25 mM DTT, and 10 mM Na VO (Sigma-Aldrich, St. Louis, MO). Homogenized liver samples were lysed on ice for 20 minutes and centrifuged at 12.000 × g for 2 minutes at 4 °C. After centrifugation the supernatant was transferred to new vials and stored at −80 °C until analysis. The protein concentration was measured spectrophotometrically (Bio-Rad Protein Assay Dye Reagent Concentrate, Bio-Rad Laboratories GmbH, München, Germany). A total of 60 μg protein was mixed with LDS Sample buffer and Sample Reducing Agent (Life Technologies, Carlsbad, CA) prior to separation on an SDS-PAGE 4–12% Bis-Tris gel (Life Technologies, Carlsbad, CA). The gels were run in MES SDS Running Buffer in the XCell SureLock Mini-Cell system and blotted using the iBlot equipment (Life Technologies, Carlsbad, CA) according to manufacturer’s instruction and as described previously^31^. As primary antibody we used the rabbit anti-V5 antibody (diluted (1:1000) (Sigma-Aldrich, St. Louis, MO) and the secondary goat anti-rabbit HRP antibody (diluted 1:5000) (DakoCytomation, Glostrup, Denmark). Protein bands was visualized using the Pierce^®^ ECL 2 Western Blotting Substrate (Thermo Fisher Scientific, Rockford, IL) according to manufacturer’s instructions. Chemoluminescence signals were detected using the G:Box Chemi XX6 gel documentation system (Syngene, Cambridge, UK).

### Detection of IFN-γ and IL-2 producing CD8^+^ T cells by ELISpot assay

Secretion of mouse IFN-γ and IL-2 by splenocytes were detected using a commercial ELISpot assay (Mabtech, Nacka Strand, Sweden). Plates (96-well, IP Sterile White Plates, 0.45 μm Hydrophobic High Protein Binding Immobilon-P Membrane, Merck Millipore, Billerica, MA) were coated with anti-IFN-γ (AN18) or anti-IL-2 (1A12) in PBS over night at 4 °C. Thereafter plates were washed with PBS and blocked using complete CTL medium (RPMI 1640 medium supplemented with 10% fetal bovine serum (FBS), 1 mM sodium pyruvate, 10 mM HEPES buffer, 1 mM nonessential amino acids, 50 mM 2-mercaptoethanol, 100 U/ml penicillin, and 100 μg/ml streptomycin [Life Technologies, Carlsbad, CA]) for 2 hours at 37 °C with 5% CO_2_. A single cell suspension was prepared by squeezing the spleen through a cell strainer (70 μm, BD Biosciences, San Jose, CA) with the back of a syringe. The single cell suspensions were lysed with Red Blood Cell Lysing Buffer (Sigma-Aldrich, St. Louis, MO) to exclude red blood cells. Thereafter, the cell suspension was re-suspended in complete CTL medium and counted. Plates were seeded with 200.000 cells in each well and mixed with the respective antigens. Seeded plates were incubated at 37 °C with 5% CO_2_ for 24 hours (IL-2) and 48 hours (IFN-γ). After incubation, the plates were washed with PBS and developed by addition of a secondary antibody (anti-IL-2 5H-4 Biotin) or (anti-IFN-γ RA-6A2 Biotin) for 1 hour. Thereafter the plates were washed again with PBS, and subsequently addition of strep-ALP for 1 hour. Spots were developed using the BCIP/NBT Plus development solution (Mabtech, Nacka Strand, Sweden) for 15 minutes (IL-2) and 12 minutes (IFN-γ). Plates were extensively washed with water to stop the reaction. The number of spots was counted using the AID iSpot reader and software version 7.0 (AID, Strassberg, Germany. The number of spots (cytokine producing cells) was determined at each concentration of peptide or protein and the results given as the number of IFN-γ or IL-2 producing cells per 10^6^ cells. A mean number of cytokine producing cells of 50 per 10^6^ cells were considered as negative.

### T cell differentiation and TCR gene transfer by electroporation

PBMCs were collected under informed consent from healthy donors. PBMCs were stimulated with 600 U/ml IL-2 (rIL-2; R&D Systems, Minneapolis, MN) and 50 ng/ml anti-CD3 (OKT-3; eBioscience, San Diego, CA) in AIM-V medium (Life Technologies, Carlsbad, CA) supplemented with 2% human AB serum for 7 days. The concentration of rIL-2 was increased to 1,000 IU/ml on day 8.

Electroporation were performed with the Nucleofector device II (Lonza, Cologne, Germany). 10 × 10^6^ PBMCs were activated for 8 days as described above and were thereafter re-suspended in 100 μL of Cell Line Nucleofector Solution V (Lonza, Cologne, Germany) and TCR mRNA was added at 200 μg/ml^47^. The mixture was placed in a certified cuvette (Lonza, Cologne, Germany) and electroporated. After electroporation, cells were re-suspended in AIM-V medium (Life Technologies, Carlsbad, CA) supplemented with 2% human AB serum and 100 IU/ml rIL-2. Transfected cells were maintained in a humidified 37 °C and 5% CO_2_ incubator until flow cytometry analysis and/or co-culture experiments.

### Functional analysis of TCR-redirected T cells

Twenty-four hours after TCR mRNA transfection, the T cells were stained with antibodies for CD3, CD8 and TCRVβ (BD Biosciences, San Jose, CA) of the respective TCR and analyzed by a FACSVerse flow cytometer (BD Biosciences, San Jose, CA). Dead cells were excluded by LIVE/DEAD Aqua dye (Life Technologies, Carlsbad, CA). The data was analyzed with the FlowJo V10.0.7 software (Tree Star, Ashland, OR). The NS3 H4 and F8 TCRs, and the NS5A 69 and 19 TCRs were analyzed. Redirected T cells were thereafter co-cultured with gt1a NS3_1073-1081_ peptide- or gt1b NS5A_1992-2000_ peptide-loaded T2 cells overnight. IFN-γ secretion into supernatants was quantified using the IFN-γ ELISA kit (Mabtech, Nacka Strand, Sweden).

Antiviral efficacy in the respective anti-HCV TCRs were measured as previously described^30^ in co-culture assays with Huh7^A2^HCV^Rep^ cells that contains a stably replicating HCV reporter replicon. The replicon-driven luciferase activity was measured using the ONE-Glo Luciferase Assay System (Promega, Madison, WI). Data is presented as percentage of relative light unit (RLU) reduction compared to untreated Huh7^A2^HCV^Rep^ cells alone (without co-culture). RLU values of non-luciferase cells (transfected T cells alone) were subtracted.

### Quantification of HCV-specific CD8^+^ T cells

Quantification of HCV NS3- and NS5A-specific CD8^+^ T cells was performed as described previously^17^. In brief, splenocytes were harvested and used directly *ex vivo* for quantification of NS3- and NS5A-specific CD8^+^ T cells. One million splenocytes were washed and suspended in FACS buffer (PBS/1% FBS). The cells were incubated with R-PE–labeled H-2D^b^ (NS3) and H-2K^b^ (NS5A) Pro5 pentamer refolded with HCV NS3_1629-1637_ (GAVQNEVTL) or HCV NS5A_2251-2259_ (VILDSFDPL) at 22 °C for 15 minutes. After pentamer labeling the cells were blocked with Fc block (anti-mouse CD16/32) on ice for 15 minutes and then stained with surface markers as follows: anti-mouse CD19-PE-Cy5 (clone 6D5) or CD19-APC (clone 1D3) and CD8-FITC (clone KT15) on ice for 20 minutes. All antibodies and pentamers were purchased from ProImmune (Oxfords, UK) except CD19-APC that was purchased from BD Biosciences (San Jose, CA). Cells were then fixed using 2% paraformaldehyde diluted in PBS. FACSCalibur or FACSVerse (BD Biosciences) were used to acquire 150,000 events and thereafter the results were analyzed with BD Biosciences CellQuest (San Jose, CA) or FlowJo 9.2 software (Ashland, OR). Living lymphocytes were gated, and from this gate the CD19^+^ cells were excluded, and further gating on CD8^+^ and Pentamer^+^ cells were performed to determine the frequency of NS3- or NS5A-specific CD8^+^ T cells.

### Immunohistochemistry

Liver pieces from two lobes were collected and fixed in 4% zinc paraformaldehyde solution (HistoLab, Gothenburg, Sweden) overnight. The paraformaldehyde-fixed liver samples were embedded in paraffin and sectioned at 4 μm and mounted on a glass slide. The slides were deparaffinized and rehydrated (xylene, 99,5% EtOH, 95% EtOH, 70% EtOH, deionized water) and thereafter incubated in a pressure-cooker (V5: antigen retrieval solution in 0.01 M citrate buffer pH 6, CD3; antigen unmasking solution pH 9 [H-3301, Vector Laboratories, Burlingame, CA]). The slides were cooled down and rinsed with deionized water followed by PBS and then addition of blocking solution (2,5% normal horse serum [MP-7401, Vector Laboratories, Burlingame, CA]). After incubation, the primary antibody solution was added (V5; goat anti-V5 [V8137, Sigma Aldrich, St. Louis, MO], CD3; polyclonal rabbit anti-human CD3 [A0452, Dakocytomation, Agilent Technologies, Santa Clara, CA]). The slides were rinsed in PBS and deionized water and then incubated in 3% H_2_O_2_ to block endogenous peroxidase activity. Slides were rinsed in PBS followed by incubation with DAB (ImmPACT DAB Peroxidase substrate kit, SK-4105, Vector Laboratories, Burlingame, CA). Slides were rinsed in deionized water and incubated in Mayers HTX (HistoLab, Gothenburg, Sweden). Slides were rinsed until the nuclei turn blue and thereafter treated with 70% EtOH, 95% EtOH, 99,5% EtOH, xylene and thereafter mounted with cover glass. A standard light microscope was used to count the slides at 400x magnification. For NS5A an area of 15 mm^2^ and for CD3 an area of 2.5 mm^2^ was counted.

### *In vivo* bioluminescence imaging

Visualization of HCV NS5A-luciferase reporter gene expression was performed in anaesthetized mice using the IVIS Spectrum *in vivo* imaging system (Xenogen IVIS® Spectrum, Caliper Life Sciences, Hopkinton, MA). To detect the presence of reporter gene expression, mice were shaved and injected with 15 mg/kg body weight luciferin substrate (D-Luciferin, K^+^ salt, PerkinElmer, Waltham, MA) diluted in 200 μL 4 minutes before anaesthetized with isofluran (IsoFlo^®^, Abbott Laboratories Ltd, Berkshire, UK). Mice were analyzed in the IVIS machine 11 minutes after the luciferin injection. Images and assessment of emitted light were analyzed with the Living Image Software version 4.2.

### Statistical analysis

Area under the curve (AUC) calculations was performed using Microsoft Excel 2011 for Macintosh (version 14.4.5; Microsoft, Redmond, WA). Statistical analysis of parametrical data was compared using the analysis of variance (ANOVA) (GraphPad InStat 3, Macintosh, version 3.0b, 2003; GraphPad Software, San Diego, CA) and non-parametrical data with Mann-Whitney U test (Prism, Macintosh, version 5.0f).

## Additional Information

**How to cite this article**: Holmström, F. *et al*. Functional differences in hepatitis C virus nonstructural (NS) 3/4A- and 5A-specific T cell responses. *Sci. Rep*. **6**, 24991; doi: 10.1038/srep24991 (2016).

## Figures and Tables

**Table 1:** Identified HCV NS5A HLA-A2.1 epitopes.

Peptide	Vaccine sequence (gt1b)	Reference sequence	Reference
NS5A-1	ILDSFDPLR	ILDSFDPLV	Cerny A *et al*.
NS5A-2	VLTDFKTWL	VLSDFKTWL	Urbani S *et al*.
NS5A-3	LLREDVTFQV	LLREEVSFRV	Urbani S *et al*.
NS5A-4	SPDADLIEANL	SPDAELIEANL	Wong DKH *et al*.

Peptide sequences of the currently known NS5A HLA-A2.1 epitopes. The table shows vaccine epitope-sequences and the respective PubMed reference.

**Table 2:** Polymorphism of HCV NS5A HLA-A2.1 epitopes.

GenBank acc. No.	Amino acid sequence	Amino acid sequence	Amino acid sequence	Amino acid sequence
Vaccine	ILDSFDPLR	VLTDFKTWL	LLREDVTFQV	SPDADLIEANL
	- - - - - - - - L	- - S - - - - - -	- - - - E - S - R -	- - - - E - - - - - -
ABH10009.1	- - - - - - - - -	- - - - - - - - -	- - - D E - - - - -	- - G - - - - - - - -
AAQ86584.1	- - - - - - - - -	#	#	- - - - - - - - - - -
AAQ86575.1	- - - - - - - - -	#	#	- - - - - - - - - - -
ACX46407.1	- - - - - - - - -	#	#	- - - - - - - - - - -
AFV79735.1	- - - - - - - - -	- - - - - - - - -	- - - - E - - - - -	- - - - - - V - - - -
AAM81351.1	- - - - - - - - -	#	#	- - - - - - - - - - -
ACJ37239.1	- - - - - - - - -	#	#	- - - - - - - - - - -
AHB33171.1	- - - - - - - - Q	- - - - - - - - -	- - - - - - - - - -	- - - - - - - - - - -
AAC41017.1	- - - - - - - - -	- - - - - - - - -	#	- - - - - - - - - - -
AFX76968.1	- - - - - - - - -	- - - - - - - - -	- - - - E - - - - -	- - - - - - - - - - -
AFX76792.1	- - - - - - - - -	- - - - - - - - -	- - - - E - - - L -	- - - - - - - - - - -
ACA49631.1	- - - - - - - - -	- - - - - - - - -	- - - D E - - - - -	- - - - - - - - - - -
AAK26363.1	#	#	#	- - - - - - - - - - -
ABF50034.1	- - - - - - - - -	- - - - L - - - -	- - - - E - - - - -	- - - - - - - - - - -
ABF48716.1	- - - - - - - - -	#	#	- - - V - - V - - - -
BAL49564.1	#	- - - - - - - - -	- - - D E - - - - -	#
AAB87527.2	- - - - - - - - H	#	- - - D E - V - - -	- - - - - - - - - - -
AAV83505.1	- - - - - - - - -	- - A - - - - - -	- - - - E - - - L -	- - - - - - - - - - -
CAL46827.1	- - - - - - - - V	#	- - - D E - S - R -	- - - - E - - A - - -
AAG22658.2	- - - - - - - - V	- - S - - - - - -	- - - D E - S - R -	- - - - E - - - - - -
AAG22647.2	- - - - - - - - V	- - S - - - - - -	- - - D E - S - R -	- - - - E - - - - - -

Polymorphism within HCV NS5A HLA-A2.1 epitopes of HCV patient isolates. An “#” indicate epitope sequences not found in the patient isolate.
